# Quantum Lernmatrix

**DOI:** 10.3390/e25060871

**Published:** 2023-05-29

**Authors:** Andreas Wichert

**Affiliations:** Department of Computer Science and Engineering, INESC-ID & Instituto Superior Técnico, University of Lisbon, 2740-122 Porto Salvo, Portugal; andreas.wichert@tecnico.ulisboa.pt

**Keywords:** Lernmatrix, associative memory, quantum counting, quantum search algorithms, qiskit

## Abstract

We introduce a quantum Lernmatrix based on the Monte Carlo Lernmatrix in which *n* units are stored in the quantum superposition of $\log_{2}\left(n\right)$ units representing $O\left(\frac{n^{2}}{\log{\left(n\right)}^{2}}\right)$ binary sparse coded patterns. During the retrieval phase, quantum counting of ones based on Euler’s formula is used for the pattern recovery as proposed by Trugenberger. We demonstrate the quantum Lernmatrix by experiments using *qiskit*. We indicate why the assumption proposed by Trugenberger, the lower the parameter temperature *t*; the better the identification of the correct answers; is not correct. Instead, we introduce a tree-like structure that increases the measured value of correct answers. We show that the cost of loading *L* sparse patterns into quantum states of a quantum Lernmatrix are much lower than storing individually the patterns in superposition. During the active phase, the quantum Lernmatrices are queried and the results are estimated efficiently. The required time is much lower compared with the conventional approach or the of Grover’s algorithm.

## 1. Introduction

There are two popular models of quantum associative memories, the quantum associative memory as proposed by Venture and Martinez [1,2,3] and the quantum associative memory as proposed by Trugenberger [4,5]. Both models store binary patterns represented by linear independent vectors by basis encoding. They prepare the linear independent states by a procedure that is based on dividing present superposition into processing and memory terms flagged by an ancilla bit. New input patterns are successively loaded into the processing branch that is divided by a parametrized controlled-U operation on an ancilla and then the pattern is merged, resulting in a superposition of linear independent states. The method is linear in the number of stored patterns and their dimension [6].

In the quantum associative memory as proposed by Venture and Martinez, a modified version of Grover’s search algorithm [7,8,9,10], ref. [7] is applied to determine the answer vector to a query vector [1,2,3]. In Trugenberger’s model, the retrieval mechanism is based on Euler’s formula to determine if the input pattern is similar to the set of stored patterns. In an additional step, the most similar pattern can be estimated by the introduced temperature parameter or alternatively by the Grover’s search algorithm. Both models suffer from the problem of input destruction (**ID problem**) [11,12,13]:

- The input (reading) problem: The amplitude distribution of a quantum state is initialized by reading *n* data points. Although the existing quantum algorithm requires only $O(\sqrt{n})$ steps or less and is faster than the classical algorithms, *n* data points must be read. Hence, the complexity of the algorithm does not improve and is $O\left(n\right)=O\left(n\right)+O\left(\sqrt{n}\right)$.
- The destruction problem: A quantum associative memory [1,2,3,4,5] for *n* data points for dimension *m* requires only m·log(n) or fewer units (quantum bits). An operator, which acts as an oracle [3], indicates the solution. However, this memory can be queried only once because of the collapse during measurement (destruction); hence, quantum associative memory does not have any advantages over classical memory.

Most quantum machine learning algorithms suffer from the input destruction problem [13]. Trugenberger tries to overcome the destruction problem by the probabilistic cloning of the quantum associative memory [4,14]. This approach was criticized in [15]. The efficient preparation of data is possible in part for spare data [16]. However, the input destruction problem is not solved till today, and usually theoretical speed ups are analyzed [17] by ignoring the input problem, which is the main bottleneck for data encoding.

In our approach, the preparation costs in which data points must be read and the query time are represented by two phases that are analyzed independently. As in the Harrow [16] approach, our data are sparse. The sparse data are stored in the best possible distributed compression methods [18,19] by a Lernmatrix [20,21] also called Willshaw’s associative memory [22]. Our quantum Lernmatrix model is based on the Lernmatrix.

We prepare a set of quantum Lernmatrices in superposition. This preparation requires a great deal of time and we name it the *sleep  phase*. On the other hand, in the *active phase*, the query operation is extremely fast. The cost of the *sleep phase* and the *active phase* are the same as one of a conventional Lernmatrix. We assume that in the *sleep phase* we have enough time to prepare several quantum Lernmatrices in superposition.

The quantum Lernmatrices are kept in superposition until they are queried in the *active phase*. Each of the copies of the quantum Lernmatrix can be queried only one time due to the destruction problem. We argue that the advantage to conventional associative memories is present in the *active phase* where the fast determination of information is essential. The naming of the phases is in analogy to a living organism that prepares itself during the sleep for an active day.

The quantum Lernmatrix does not store independent vectors, but units that represent the compressed binary patterns. The units are described by binary weight vectors that can be correlated, so we cannot use the approach as proposed by Venture, Martinez and Trugenberger. Instead, we prepare the superposition of the weight vectors of the units by the entanglement of index qubits in superposition with the weight vectors. The retrieval phase is based on Euler’s formula as suggested by Trugenberger [4,14]. However, we do not determine the Hamming distance to the query vector, but the number of ones of the query vector that are present in the weight vector. We indicate the quantum Lernmatrix *qiskit* implementation step by step. *Qiskit* is an open-source software development kit (SDK) for working with quantum computers at the level of circuits and algorithms from IBM [23]. The paper is organized as follows:

- We introduce the Lernmatrix model described by units that model neurons.
- We indicate that Lernmatrix has a tremendous storage capacity, much higher than most other associative memories. This is valid for sparse equally distributed ones in vectors representing the information.
- Quantum counting of ones based on Euler’s formula is described.
- Based on the Lernmatrix model, a quantum Lernmatrix is introduced in which units are represented in superposition and the query operation is based on quantum counting of ones. The measured result is a position of a one or zero in the answer vector.
- We analyze the Trugenberger amplification.
- Since a one in the answer vector represents information, we assume in that we can reconstruct the answer vector by measuring several ones, taking for granted that the rest of the vector is zero. In a sparse code with *k* ones, *k* measurements of different ones reconstruct the binary answer vector. We can increase the probability of measuring a one by the introduced tree-like structure.
- The Lernmatrix can store much more patterns then the number of units. Because of this, the cost of loading *L* patterns into quantum states is much lower than storing the patterns individually.

## 2. Lernmatrix

Associative memory models human memory [24,25,26,27,28]. The associative memory and distributed representation incorporate the following abilities in a natural way [18,28,29,30]:

- The ability to correct faults if false information is given.
- The ability to complete information if some parts are missing.
- The ability to interpolate information. In other words, if a sub-symbol is not currently stored, the most similar stored sub-symbol is determined.

Different associative memory models have been proposed over the years [19,28,31,32,33]. The Hopfield model represents a recurrent model of the associative memory [29,31,34], it is a dynamical system that evolves until it has converged to a stable state. The Lernmatrix, or  Willshaw’s associative memory, also simply called “associative memory” (if no confusion with other models is possible [32,33]), it was developed by Steinbuch in 1958 as a biologically inspired model from the effort to explain the psychological phenomena of conditioning [20,21]. The goal was to produce a network that could use a binary version of Hebbian learning to form associations between pairs of binary vectors. Later, this model was studied under biological and mathematical aspects mainly by Willshaw [22] and Palm [18,24] and it was shown that this simple model has a tremendous storage capacity.

**Figure 1:**
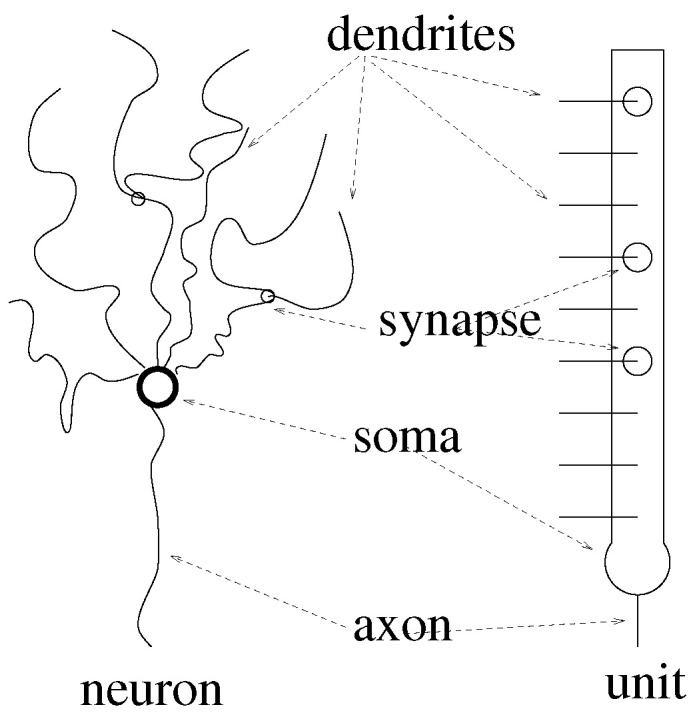
A unit is an abstract model of a biological neuron [24,29,35,36,37].

Lernmatrix is composed of a cluster of units. Each unit represents a simple model of a real biological neuron. Each unit is composed of binary weights, which correspond to the synapses and dendrites in a real neuron (see Figure 1).

**Figure 2:**
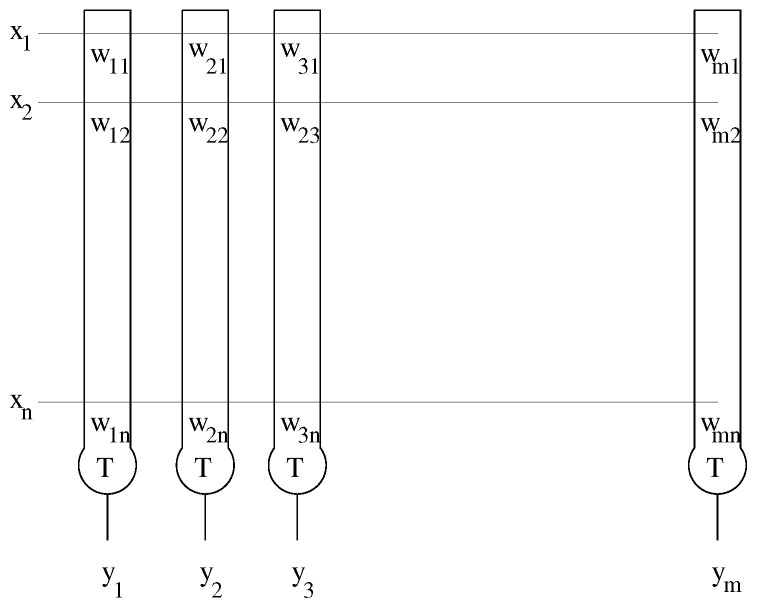
The Lernmatrix is composed of a set of units that represent a simple model of a real biological neuron. The unit is composed of weights, which correspond to the synapses and dendrites in the real neuron. In this Figure, they are described by $w_{ij}\in \left\{0,1\right\}$ where 1≤i≤m and 1≤j≤n. *T* is the threshold of the unit.

They are described by $w_{ij}\in \left\{0,1\right\}$ in Figure 2. *T* is the threshold of the unit.

The presence of a feature is indicated by a “one” component of the vector, its absence through a “zero” component of the vector. A pair of these vectors is associated and this process of association is called learning. The first of the two vectors is called the *query vector* and the second, the *answer vector*. After learning, the query vector is presented to the associative memory and the answer vector is determined by the retrieval rule.

### 2.1. Learning and Retrieval

Initially, no information is stored in the associative memory. Because the information is represented in weights, all unit weights are initially set to zero. In the learning phase, pairs of binary vector are associated. Let x be the query vector and y the answer vector, the learning rule is:

(1) $$w_{ij}^{new}=\left\{\begin{array}{cc} 1 & if\;\;y_{i}\cdot x_{j}=1\\ w_{ij}^{old} & \mathrm{otherwise}. \end{array}\right.$$

This rule is called the binary Hebbian rule [18]. Every time a pair of binary vectors is stored, this rule is used.

In the *one-step* retrieval phase of the associative memory, a fault tolerant answering mechanism recalls the appropriate answer vector for a query vector x.

The retrieval rule for the determination of the answer vector y is:

(2) $$net_{i}=\sum_{j=1}^{n}w_{ij}x_{j},$$

$$y_{i}=\left\{\begin{array}{cc} 1 & \mathrm{if}\;\;net\geq \;T\\ 0 & \mathrm{otherwise}. \end{array}\right.$$

where *T* is the threshold of the unit. The threshold *T* is set to the number of “one” components in the query vector x, T:=|x|. If the output of the unit is 1, we say that the units fires, and for the output 0 the unit does not fire. The cost of the *one-step* retrieval is O(n·m). The retrieval is called:

- Hetero-association if both vectors are different x≠y,
- Association, if x=y, the answer vector represents the reconstruction of the disturbed query vector.

For simplicity, we assume that the dimension of the query vector and the answer vector are the same, n=m.

#### Example

**Figure 3:**
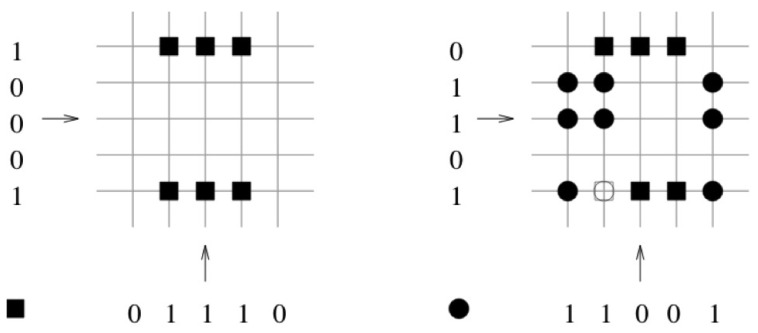
The vector pair $x_{1}=\left(1,0,0,0,1\right)$ and $y_{1}=\left(0,1,1,1,0\right)$ is learned. The corresponding binary weights of the associated pair are indicated by a black square. In the next step, the vector pair $x_{2}=\left(0,1,1,0,1\right)$ and $y_{2}=\left(1,1,0,0,1\right)$ is learned. The corresponding binary weights of the associated pair are indicated by a black circle.

**Figure 4:**
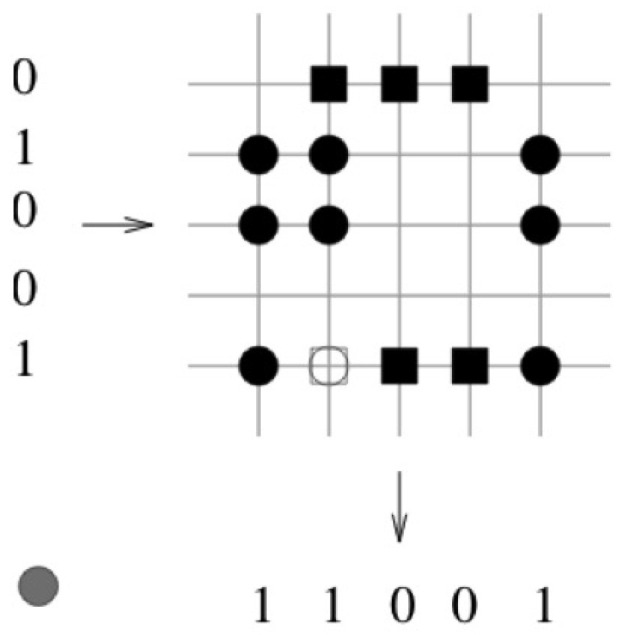
The query vector $x_{q}=\left(0,1,0,0,1\right)$ differs by one bit to the learned query vector $x_{2}=\left(0,1,1,0,1\right)$. The threshold *T* is set to the number of “one” components in the query vector $x_{q}$, T=2. The retrieved vector is the vector $y_{2}=\left(1,1,0,0,1\right)$ that was stored.

In Figure 3 the vector pair $x_{1}=\left(1,0,0,0,1\right)$ and $y_{1}=\left(0,1,1,1,0\right)$ is learned. The corresponding binary weights of the associated pair are indicated by a black square. In the next step, the vector pair $x_{2}=\left(0,1,1,0,1\right)$ and $y_{2}=\left(1,1,0,0,1\right)$ is learned. The corresponding binary weights of the associated pair are indicated by a black circle. In the third step, the retrieval phase is preformed (see Figure 4). The query vector $x_{q}=\left(0,1,0,0,1\right)$ differs by one bit to the learned query vector $x_{2}=\left(0,1,1,0,1\right)$. The threshold *T* is set to the number of “one” components in the query vector $x_{q}$, T=2. The retrieved vector is the vector $y_{2}=\left(1,1,0,0,1\right)$ that was stored.

### 2.2. Storage Capacity

We analyze the optimal storage costs of the Lernmatrix. For an estimation of the asymptotic number *L* of vector pairs (x,y) that can be stored in an associative memory before it begins to make mistakes in the retrieval phase, it is assumed that both vectors have the same dimension *n*. It is also assumed that both vectors are composed of *k* ones, which are equally likely to be in any coordinate of the vector. In this case, it was shown [18,19,38] that the optimum value for *k* is approximately

(3) $$k≐\log_{2}\left(n/4\right).$$

For example, for a vector of the dimension *n* = 1,000,000, only k=18 ones should be used to code a pattern according to the Equation (3). For an optimal value for *k* according to the Equation (3) with ones equally distributed over the coordinates of the vectors, approximately *L* vector pairs can be stored in the associative memory [18,19]. *L* is approximately

(4) $$L≐\left(\ln2\right)\left(n^{2}/k^{2}\right).$$

This value is much **greater** than *n*. The estimate of *L* is very rough because Equation (3) is only valid for very large networks; however, the capacity increase is still considerable. The upper bound for large *n* is

(5) $$I=n^{2}\log2=n^{2}\cdot 0.693$$

the asymptotic capacity is 69.311% percent per bit, which is much higher than most associative memories. This capacity is only valid for sparse equally distributed ones [18]. The promise of Willshaw’s associative memory that it can store much more patterns then the number of units. The cost of loading $L=\left(\ln2\right)\left(n^{2}/k^{2}\right)$ patterns in *n* units with $k=\log_{2}\left(n/4\right)$ is $O(n^{2})$. It is much lower than storing the *L* patterns in a list of *L* units O(n·L) This is because L>n, or 

$$O\left(\frac{n^{2}}{\log{\left(n\right)}^{2}}\right)>O\left(n\right)$$

since

$$\sqrt{n}>\log\left(n\right).$$

The Lernmatrix has a tremendous storage capacity [18,19], it can store much more patterns then the number of units.

The description of how to generated efficiently binary sparse codes of visual patterns or other data structure is described in [39,40,41]. For example, real vector patterns have to be binarized.

The asymptotic capacity is 69.311% per bit, which is much higher than most associative memories. This capacity is only valid for sparse equally distributed ones [18]. The description of how to generate efficiently binary sparse codes of visual patterns or other data structures is described in [39,40,41]. For example, real vector patterns have to be binarized.

### 2.3. Large Matrices

**Figure 5:**
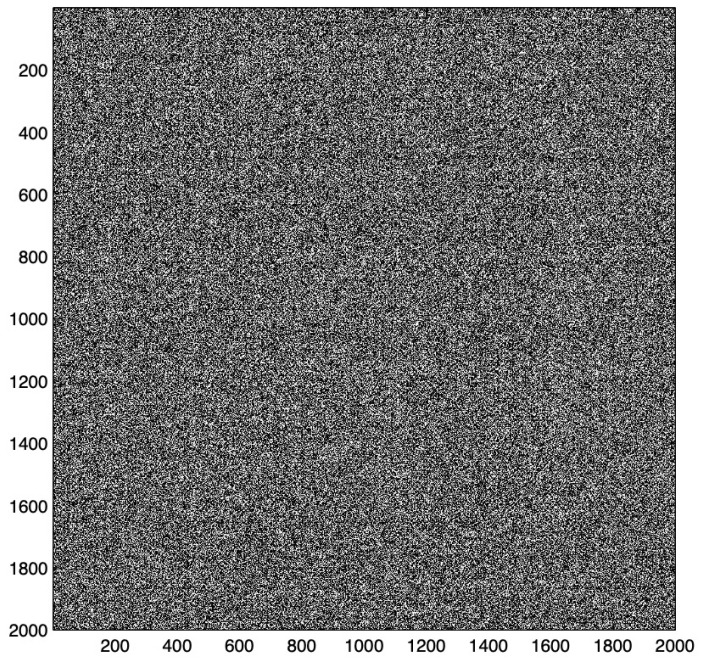
The weight matrix after learning of 20,000 test patterns, in which ten ones were randomly set in a 2000 dimensional vector represents a high loaded matrix with equally distributed weights. This example shows that the weight matrix diagram often contains nearly no information. Information about the weight matrix can be extracted by the structure of weight matrix. (White color represents wights.)

The diagram of the weight matrix illustrates the weight distribution, which results from the distribution of the stored patterns [42,43]. Useful associative properties result from equally distributed weights over the whole weight matrix and are only present in large matrices. A high percentage indicates an overload and the loss of its associative properties. Figure 5 represents a diagram of a high loaded matrix with equally distributed weights.

## 3. Monte Carlo Lernmatrix

The suggested probabilistic retrieval rule for the determination of the answer vector y for the query vector x is

(6) $$p\left(y_{i}=1|x\right)=\frac{1}{n}\cdot \left(\frac{net_{i}}{\sum_{v=1}^{n}net_{v}}\right)$$

and

(7) $$p\left(y_{i}=0|x\right)=\frac{1}{n}\cdot \left(1-\frac{net_{i}}{\sum_{v=1}^{n}net_{v}}\right)$$

describing the probability of firing or not firing of one unit with

(8) $$1=\sum_{i=1}^{n}\left(p\left(y_{i}=1|x\right)+p\left(y_{i}=0|x\right)\right).$$

During the query operation one unit is randomly sampled and either it fires or not according to the probability distribution. To determine the answer vector, we have to sample the Monte Carlo Lernmatrix several times. For the reconstructed vector three states will be present: 1 for fired units, 0 for not fired units and unknown for silent units. The Monte Carlo Lernmatrix is a close description of the quantum Lernmatrix. In the quantum Lernmatrix, units are represented by quantum states, with sampling correspond to the measurement.

## 4. Qiskit Experiments

*Qiskit* is an open-source software development kit (SDK) for working with quantum computers at the level of circuits and algorithms [23], IBM Quantum, https://quantum-computing.ibm.com/ (accessed on 25 May 2023 ), 2023, *Qiskit* (Version 0.43.0). *Qiskit* provides tools for creating and manipulating quantum programs and running them on prototype quantum devices on the IBM Quantum Experience or on simulators on a local computer. It follows the quantum circuit model for universal quantum computation and can be used for any quantum hardware that follows this model. *Qiskt* provides different backend simulator functions.

In our experiments, we use the *statevector simulator*. It performs an ideal execution of *qiskit* circuits and returns the final state vector off the simulator after application (all qubits). The state vector of the circuit can represent the probability values that correspond to the multiplication of the state vector by the unitary matrix that represents the circuit. We use the *statevector simulator* to check the value of all qubits.

If we want to simulate an actual device of today, which is prone to noise resulting from decoherence, we can use the *qasm simulator*. It returns counts, which are a sampling of the measured qubits that have to be defined in the circuit. One can easily port the simulation using

simulator=Aer.get_backend(‘statevector_simulator’)

and the command qc.measure(qubits,c) indicates that we measure the qubits (the counting begins with zero and not one) and store the result of the measurement in the conventional bits *c*.

Our description involve simple quantum circuits using basic quantum gates that can be easily ported to other quantum software development kits.

## 5. Quantum Counting Ones

**Figure 6:**
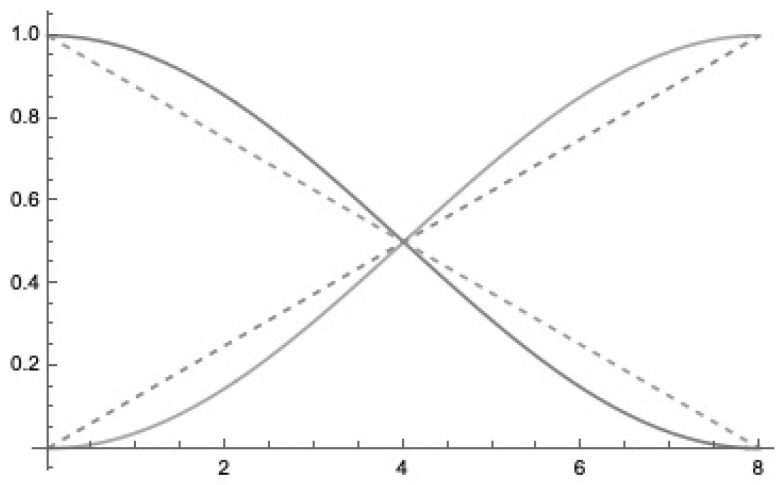
Sigmoid-like probability functions for N=8 is indicated by continuous line, the linear relation by the dashed lines. The x-axis indicates the *k* values, and the y-axis the probabilities.

In a binary string of the length *N*, we can represent the fraction of *k* ones by the simple formula k/N and of the zeros as (N−k)/N resulting in a linear relation. We can interpret these numbers as probability values. We can map these linear relations into the sigmoid-like probability functions for the presence of ones using Euler’s formula [4] in relation to trigonometry

(9) $${\left(\sin\left(\frac{\pi \cdot k}{2\cdot N}\right)\right)}^{2}={\left|\frac{e^{i\cdot \frac{\pi \cdot k}{2\cdot N}}-e^{-i\cdot \frac{\pi \cdot k}{2\cdot N}}}{2}\right|}^{2}\in \left[0,1\right]$$

and of zeros with

(10) $${\left(\cos\left(\frac{\pi \cdot k}{2\cdot N}\right)\right)}^{2}={\left|\frac{e^{i\cdot \frac{\pi \cdot k}{2\cdot N}}+e^{-i\cdot \frac{\pi \cdot k}{2\cdot N}}}{2}\right|}^{2}\in \left[0,1\right]$$

together with

$${\left(\sin\left(\frac{\pi \cdot k}{2\cdot N}\right)\right)}^{2}+{\left(\cos\left(\frac{\pi \cdot k}{2\cdot N}\right)\right)}^{2}=1$$

in the Figure 6, the sigmoid-like probability functions for N=8 are indicated.

This operation can be implemented by quantum counting of ones. In our example, the state |101〉 is represented by N=3 qubits, of which two (k=2) are one.

To count the number of ones, we introduced the control qubit in superposition $1/\sqrt{2}\cdot (|0\rangle +\left|1\rangle \right)$. For the superposition part represented by the control qubit 0, the phase $e^{i\cdot \frac{\pi }{2\cdot 3}}$ is applied for each one. For the superposition part represented by the control qubit 1, the phase $e^{-i\cdot \frac{\pi }{2\cdot 3}}$ is applied for each one.

$$\frac{1}{\sqrt{2}}\cdot |0\rangle ⊗\left(e^{i\cdot \frac{\pi }{2\cdot 3}}\cdot |1\rangle ⊗|0\rangle ⊗e^{i\cdot \frac{\pi }{2\cdot 3}}\cdot |1\rangle \right)+$$

(11) $$\frac{1}{\sqrt{2}}\cdot |1\rangle ⊗\left(e^{-i\cdot \frac{\pi }{2\cdot 3}}\cdot |1\rangle ⊗|0\rangle ⊗e^{-i\cdot \frac{\pi }{2\cdot 3}}\cdot |1\rangle \right)=$$

$$\frac{e^{i\cdot \frac{\pi \cdot 2}{2\cdot 3}}}{\sqrt{2}}|0101\rangle +\frac{e^{-i\cdot \frac{\pi \cdot 2}{2\cdot 3}}}{\sqrt{2}}|1101\rangle$$

If we apply a Hadamard gate to the control qubit [4], we obtain

$$\left(H⊗I⊗I⊗I\right)\cdot \left(\frac{e^{i\cdot \frac{\pi \cdot 2}{2\cdot 3}}}{\sqrt{2}}|0101\rangle +\frac{e^{-i\cdot \frac{\pi \cdot 2}{2\cdot 3}}}{\sqrt{2}}|1101\rangle \right)=$$

$$\frac{e^{i\cdot \frac{\pi \cdot 2}{2\cdot 3}}+e^{-i\cdot \frac{\pi \cdot 2}{2\cdot 3}}}{2}|0101\rangle +\frac{e^{i\cdot \frac{\pi \cdot 2}{2\cdot 3}}-e^{-i\cdot \frac{\pi \cdot 2}{2\cdot 3}}}{2}|1101\rangle =$$

$$\cos\left(\frac{\pi \cdot 2}{2\cdot 3}\right)\cdot |0101\rangle +i\cdot \sin\left(\frac{\pi \cdot 2}{2\cdot 3}\right)\cdot |1101\rangle =$$

(12) $$\left(\cos\left(\frac{\pi \cdot 2}{2\cdot 3}\right)\cdot |0\rangle +i\cdot \sin\left(\frac{\pi \cdot 2}{2\cdot 3}\right)\cdot |1\rangle \right)⊗|101\rangle$$

**Figure 7:**
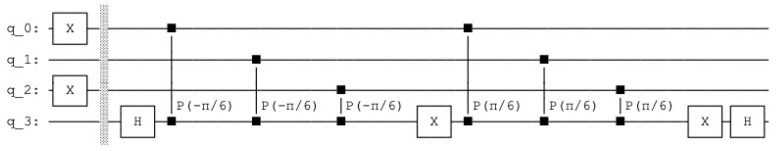
Quantum counting circuit with N=3 and k=2.

**Figure 8:**
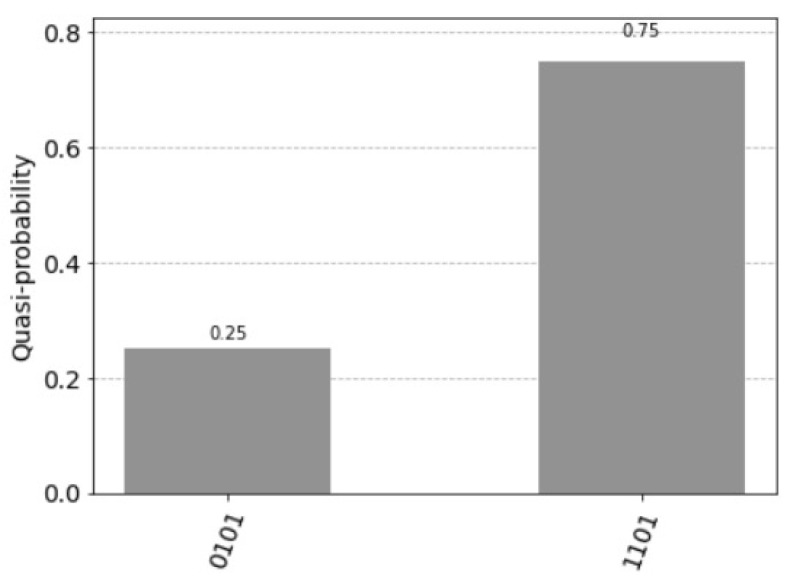
p(|0101〉)=0.25 and p(|1101〉)=0.75.

The probability of measuring the control qubit |0〉 is

$$p(|0\rangle )=p(|0101\rangle )={\left(\cos\left(\frac{\pi \cdot 2}{2\cdot 3}\right)\right)}^{2}=0.25$$

and the probability of measuring the control qubit |1〉 is

$$p(|1\rangle )=p(|1101\rangle )={\left(\sin\left(\frac{\pi \cdot 2}{2\cdot 3}\right)\right)}^{2}=0.75$$

indicating the presence of two ones. The representation of the circuit in *qiskit* is given by

from qiskit import QuantumCircuit, Aer, execute
from qiskit.visualization import plot_histogram
from math import~pi

qc = QuantumCircuit(4)
#Input is |101>
qc.x(0)
qc.x(2)
qc.barrier()
qc.h(3)
qc.cp(-pi/6,0,3)
qc.cp(-pi/6,1,3)
qc.cp(-pi/6,2,3)
qc.x(3)
qc.cp(pi/6,0,3)
qc.cp(pi/6,1,3)
qc.cp(pi/6,2,3)
qc.x(3)
qc.h(3)

simulator = Aer.get_backend(’statevector_simulator’)
# Run and get counts
result=execute(qc,simulator).result()
counts = result.get_counts()
plot_histogram(counts)

the resulting quantum circuit is represented in Figure 7 and the resulting histogram of the measured qubits is represented in Figure 8.

## 6. Quantum Lernmatrix

Useful associative properties result from equally distributed weights over the whole weight matrix and are only present in large matrices, in our examples we examine toys examples as a proof of concept for future quantum associative memories.

The superposition of the weight vectors of the units is based on the entanglement of the index qubits that are in the superposition with the weight vectors. The count is represented by a unary string of qubits that controls the phase operation. It represents the net value of the Lernmatrix. The phase information is the basis of the quantum counting of ones that increases the probability of measuring the correct units representing ones in the answer vector. We will represent *n* units in superposition by entanglement with the index qubits.

**Figure 9:**
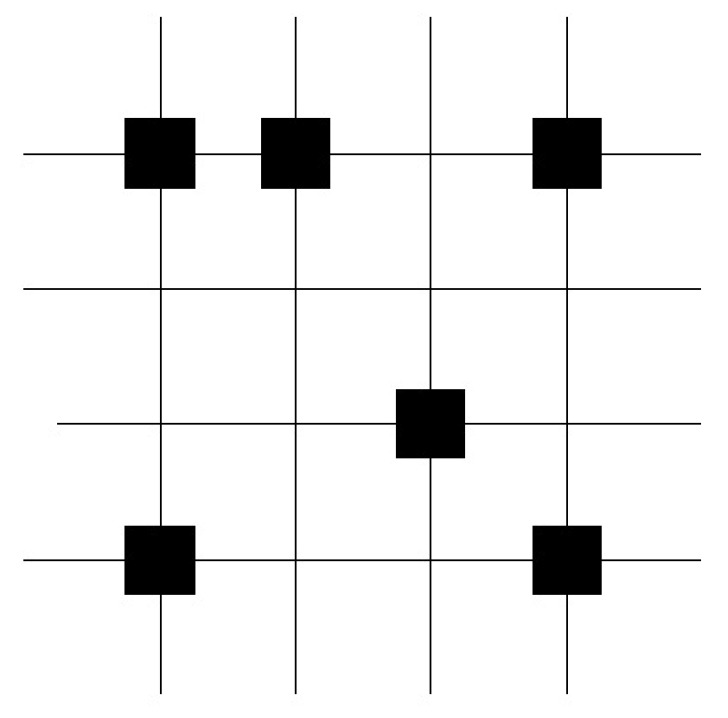
Wight matrix represented by four units after learning the correlation of the three patterns $x_{1}=\left(1,0,0,1\right)$; $y_{1}=\left(1,0,0,1\right)$, $x_{2}=\left(1,0,0,0\right)$; $y_{2}=\left(0,1,0,0\right)$ and $x_{3}=\left(0,0,1,0\right)$; $y_{3}=\left(0,0,1,0\right)$. The learning is identical with the learning phase of the Lernmatrix.

To represent four 4 units, we need two index qubits in superposition. Each index state of the qubit is entangled with a pattern by the Toffoli gate also called the ccX gate (CCNOT gate, controlled controlled not gate), by setting a corresponding qubit to one. In our example, we store three patterns $x_{1}=\left(1,0,0,1\right)$; $y_{1}=\left(1,0,0,1\right)$, $x_{2}=\left(1,0,0,0\right)$; $y_{2}=\left(0,1,0,0\right)$ and $x_{3}=\left(0,0,1,0\right)$; $y_{3}=\left(0,0,1,0\right)$ resulting in the weight matrix represented by four units (see Figure 9).

After the entanglement of index qubits $|index_{j}\rangle$ in superposition

$$|index_{1}\rangle =|11\rangle \;\;\;|index_{2}\rangle =|10\rangle$$

$$|index_{3}\rangle =|01\rangle \;\;\;|index_{4}\rangle =|00\rangle$$

with the weight vectors the following state is present, the state $count_{j}$ and $unit_{j}$ are represented by four qubits each for the four binary weights, with 

$$|unit_{j}\rangle =|{\left(w_{1}w_{2}w_{3}w_{4}\right)}_{j}\rangle$$

(see Figure 10)

With

(13) $$\frac{1}{2}\cdot \left(\sum_{j=1}^{4}|count_{j}\rangle |unit_{j}\rangle |index_{j}\rangle \right).$$

The value $|count_{j}\rangle$ is the unary representation of the Lernmatrix value $net_{j}$. We include the query vector as $x_{q}=\left(1,0,0,1\right)$,

$$\frac{1}{2}\cdot \left(\sum_{j=1}^{4}|count_{j}\rangle |unit_{j}\rangle |index_{j}\rangle \right)⊗|query\rangle =$$

(14) $$\frac{1}{2}\cdot \left(\sum_{j=1}^{4}|{\left(c_{1}c_{2}c_{3}c_{4}\right)}_{j}\rangle |{\left(w_{1}w_{2}w_{3}w_{4}\right)}_{j}\rangle |{\left(i_{1}i_{2}\right)}_{j}\rangle \right)⊗|1001\rangle$$

the resulting histogram of the measured qubits is represented in Figure 11.

In the next step, we describe the *active phase* (see Figure 12).

For simplicity, we will ignore the index qubits, since they are not important in the active phase. We perform quantum counting using the control bit that is set in superposition resulting in

$$\frac{1}{\sqrt{2}}\cdot (|0\rangle +\left|1\rangle \right)⊗\frac{1}{2}\cdot \left(\sum_{j=1}^{4}|{\left(c_{1}c_{2}c_{3}c_{4}\right)}_{j}\rangle |{\left(w_{1}w_{2}w_{3}w_{4}\right)}_{j}\rangle \right)⊗|1001\rangle =$$

$$\frac{1}{2\cdot \sqrt{2}}\cdot |0\rangle \left(\sum_{j=1}^{4}|{\left(c_{1}c_{2}c_{3}c_{4}\right)}_{j}\rangle |{\left(w_{1}w_{2}w_{3}w_{4}\right)}_{j}\rangle \right)⊗|1001\rangle +$$

(15) $$\frac{1}{2\cdot \sqrt{2}}\cdot |1\rangle \left(\sum_{j=1}^{4}|{\left(c_{1}c_{2}c_{3}c_{4}\right)}_{j}\rangle |{\left(w_{1}w_{2}w_{3}w_{4}\right)}_{j}\rangle \right)⊗|1001\rangle$$

Applying controlled phase operation with N=2 since two ones are present in the query vector and $count_{j}\leq 2$

$$\frac{1}{2\cdot \sqrt{2}}\cdot |0\rangle \left(\sum_{j=1}^{4}e^{i\cdot \frac{\pi \cdot count_{j}}{2\cdot 2}}\cdot |{\left(c_{1}c_{2}c_{3}c_{4}\right)}_{j}\rangle |{\left(w_{1}w_{2}w_{3}w_{4}\right)}_{j}\rangle \right)⊗|1001\rangle +$$

(16) $$\frac{1}{2\cdot \sqrt{2}}\cdot |1\rangle \left(\sum_{j=1}^{4}e^{-i\cdot \frac{\pi \cdot count_{j}}{2\cdot 2}}\cdot |{\left(c_{1}c_{2}c_{3}c_{4}\right)}_{j}\rangle |{\left(w_{1}w_{2}w_{3}w_{4}\right)}_{j}\rangle \right)⊗|1001\rangle$$

and applying the Hadamard gate to the control qubit, we obtain

$$\left(\sum_{j=1}^{4}\frac{1}{2}\cdot \left(\cos\left(\frac{\pi \cdot count_{j}}{2\cdot 2}\right)\right)\cdot \left|0\rangle \right|{\left(c_{1}c_{2}c_{3}c_{4}\right)}_{j}\rangle |{\left(w_{1}w_{2}w_{3}w_{4}\right)}_{j}\rangle \right)⊗|1001\rangle +$$

(17) $$\left(\sum_{j=1}^{4}\frac{1}{2}\cdot \left(i\cdot \sin\left(\frac{\pi \cdot count_{j}}{2\cdot 2}\right)\right)\cdot \left|1\rangle \right|{\left(c_{1}c_{2}c_{3}c_{4}\right)}_{j}\rangle |{\left(w_{1}w_{2}w_{3}w_{4}\right)}_{j}\rangle \right)⊗|1001\rangle .$$

The architecture is described by fifteen qubits, see Appendix A. With the query vector $x_{q}=\left(1,0,0,1\right)$ units represented by the states have following values:

- The first unit has the value $count_{1}=2$ and the two corresponding states are: for the control qubit=1 the value is $1=\sin\frac{\pi }{2}$ with the measured probability ${\left|\sin\frac{\pi }{2}\cdot \frac{1}{2}\right|}^{2}=0.25$ and for the control qubit=0 the value is $0=\cos\frac{\pi }{2}$ with the measured probability 0.
- The second unit has the value $count_{2}=1$ and the two corresponding states are: for the control qubit=1 the value is $\frac{1}{\sqrt{2}}=\sin\frac{\pi }{4}$ with the measured probability ${\left|\sin\frac{\pi }{4}\cdot \frac{1}{2}\right|}^{2}=0.125$ and for the control qubit=0 the value is $\frac{1}{\sqrt{2}}=\cos\frac{\pi }{4}$ with the measured probability ${\left|\cos\frac{\pi }{4}\cdot \frac{1}{2}\right|}^{2}=0.125$.
- The third unit has the value $count_{3}=0$ and the two corresponding states are: for the control qubit=1 the value is 0=sin0 with the measured probability = 0 and for the control qubit=0 the value is 1=cos0 with the measured probability = 0.
- The fourth unit has the (decimal) value $count_{4}=2$ and the two corresponding states are: for the control qubit=1 the value is $1=\sin\frac{\pi }{2}$ with the measured probability ${\left|\sin\frac{\pi }{2}\cdot \frac{1}{2}\right|}^{2}=0.25$ and for the control qubit=0 the value is $0=\cos\frac{\pi }{2}$ with the measured probability 0.

There are five states with probabilities not equal to zero, see Figure 13. The measured probability (control qubit=1) indicating a firing of the units is 0.625.

### 6.1. Generalization

We can generalize the description for *n* units. After  the entanglement of index qubits in superposition with the weight vectors, the following state is present, and the state $count_{j}$ and $unit_{j}$ are represented by [4,5],

(18) $$\frac{1}{\sqrt{n}}\cdot \left(\sum_{j=1}^{n}|count_{j}\rangle |unit_{j}\rangle |index_{j}\rangle \right)⊗|query\rangle .$$

with the cost $O(n^{2})$. We apply the control qubit (ignoring the index qubits)

$$\frac{1}{\sqrt{2}}\cdot (|0\rangle +\left|1\rangle \right)⊗\frac{1}{\sqrt{n}}\cdot \left(\sum_{j=1}^{n}|count_{j}\rangle |unit_{j}\rangle \right)⊗|query\rangle =$$

$$\frac{1}{\sqrt{2\cdot n}}\cdot |0\rangle \left(\sum_{j=1}^{n}|count_{j}\rangle |unit_{j}\rangle \right)⊗|query\rangle +$$

(19) $$\frac{1}{\sqrt{2\cdot n}}\cdot |1\rangle \left(\sum_{j=1}^{n}|count_{j}\rangle |unit_{j}\rangle \right)⊗|query\rangle .$$

Applying controlled phase operation with *N* for present ones in the query vector and $count_{j}\leq N$

$$\frac{1}{\sqrt{2\cdot n}}\cdot |0\rangle \left(\sum_{j=1}^{n}e^{i\cdot \frac{\pi \cdot count_{j}}{2\cdot N}}\cdot |count_{j}\rangle |unit_{j}\rangle \right)⊗|query\rangle +$$

(20) $$\frac{1}{\sqrt{2\cdot n}}\cdot |1\rangle \left(\sum_{j=1}^{n}e^{-i\cdot \frac{\pi \cdot count_{j}}{2\cdot N}}\cdot |count_{j}\rangle |unit_{j}\rangle \right)⊗|query\rangle$$

and applying the Hadamard gate to the control qubit, we obtain the final result with

$$\left(\sum_{j=1}^{n}\frac{1}{\sqrt{n}}\cdot \left(\cos\left(\frac{\pi \cdot count_{j}}{2\cdot N}\right)\right)\cdot |0\rangle |count_{j}\rangle |unit_{j}\rangle \right)⊗|query\rangle +$$

(21) $$\left(\sum_{j=1}^{n}\frac{1}{\sqrt{n}}\cdot \left(i\cdot \sin\left(\frac{\pi \cdot count_{j}}{2\cdot N}\right)\right)\cdot |1\rangle |count_{j}\rangle |unit_{j}\rangle \right)⊗|query\rangle$$

The cost of one query is O(n) and for $k=\log_{2}\left(n/4\right)$ queries O(log(n)·n).

### 6.2. Example

In this example, we store three patterns representing three associations: $x_{1}=\left(1,1,0,0,0,0,1,0\right)$; $y_{1}=\left(1,1,0,0,0,0,1,0\right)$, $x_{2}=\left(0,1,0,1,1,0,0,0\right)$; $y_{2}=\left(0,1,0,1,1,0,0,0\right)$ and $x_{3}=\left(0,0,1,0,0,1,0,1\right)$; $y_{3}=\left(0,0,1,0,0,1,0,1\right)$. The weight matrix after the learning phase is represented by eight units (see Figure 14 and Figure 15).

After the entanglement of index qubits in superposition

$$|index_{1}\rangle =|111\rangle \;\;\;|index_{2}\rangle =|110\rangle$$

$$|index_{3}\rangle =|101\rangle \;\;\;|index_{4}\rangle =|100\rangle$$

$$|index_{5}\rangle =|011\rangle \;\;\;|index_{6}\rangle =|010\rangle$$

$$|index_{7}\rangle =|001\rangle \;\;\;|index_{8}\rangle =|000\rangle$$

with the weight vectors, the following state is present, the state $count_{j}$ and $unit_{j}$ are represented by eight qubits [4,5],

$$\frac{1}{\sqrt{8}}\cdot \left(\sum_{j=1}^{8}|count_{j}\rangle |unit_{j}\rangle |index_{j}\rangle \right).$$

With the query vector as $x_{q}=\left(1,1,0,0,0,0,0,0\right)$, we obtain (see Figure 16)

$$\frac{1}{\sqrt{8}}\cdot \left(\sum_{j=1}^{8}|count_{j}\rangle |unit_{j}\rangle |index_{j}\rangle \right)⊗|11000000\rangle .$$

and the answer vector (ignoring the index qubits) according to

$$\left(\sum_{j=1}^{8}\frac{1}{\sqrt{8}}\cdot \left(\cos\left(\frac{\pi \cdot count_{j}}{2\cdot N}\right)\right)\cdot |0\rangle |count_{j}\rangle |unit_{j}\rangle \right)⊗|11000000\rangle +$$

$$\left(\sum_{j=1}^{8}\frac{1}{\sqrt{8}}\cdot \left(i\cdot \sin\left(\frac{\pi \cdot count_{j}}{2\cdot N}\right)\right)\cdot |1\rangle |count_{j}\rangle |unit_{j}\rangle \right)⊗|11000000\rangle$$

is (1,1,0,0,0,0,1,0) (see Figure 17).

## 7. Applying Trugenberger Amplification Several Times

**Figure 10:**
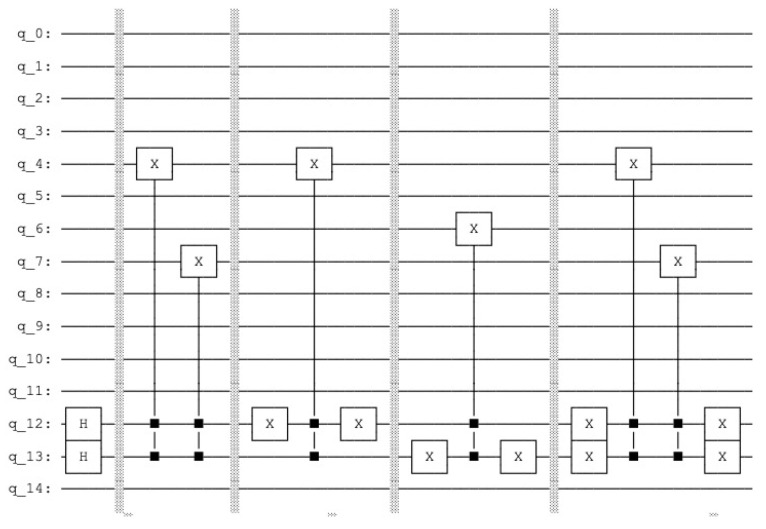
The quantum circuit that produces the *sleep phase*. The qubits 0 to 3 represent the query vector, the qubits 4 to 7 the associative memory, the qubits 8 to 11 represent the count and the qubits 12 and 13 are the index qubits, while the qubit 14 is the control qubit.

According to Trugenberger [5], applying the control qubit sequential *b* times results in

(22) $$\begin{array}{c} \sum_{v=0}^{b}\left(\sum_{j=1}^{n}\frac{1}{\sqrt{n}}\cdot {\left(\cos\left(\frac{\pi \cdot count_{j}}{2\cdot N}\right)\right)}^{b-v}\cdot {\left(i\cdot \sin\left(\frac{\pi \cdot count_{j}}{2\cdot N}\right)\right)}^{v}\cdot \right.\\ \cdot |v\rangle |count_{j}\rangle |unit_{j}\rangle |index_{j}\rangle )⊗|query\rangle . \end{array}$$

with |v〉 being the binary representation of the decimal value *v*. The idea is then to measure *b* control qubits *b* times, until the desired state is obtained. Trugenberger identifies the inverse parameter *b* as temperature t=1/b and concludes that the accuracy of pattern recall can be tuned by adjusting a parameter playing the role of an effective temperature [5]. In Figure 18, the control qubit was applied two times for the quantum circuit of the Figure 10. Figure 19 represents the resulting histogram of the measured qubits.

### Relation to b

Trugenberger [5] identifies t=1/b as a temperature and concludes: the lower *t*; the better one can identify the desired states. Assuming we have eight states indicated by the index qubit 2, 3 and 4, one marked state 010 has the count two, and the other seven state the count of one, see Figure 20. Figure 21 represents the resulting histogram of the measured qubits (b=1) and Figure 22 represents the resulting histogram after applying the control qubit two times (b=2).

Now we can take the idea further and generalize it. For *n* states, one state is marked with the count of 2, and all other remaining states have the count of 1. Since there are *n* states, the marked state has the probability value 1/n and the 2·(n−1) remaining states have the probability value p(x). It follows

(23) $$\frac{1}{n}+2\cdot \left(n-1\right)\cdot p\left(x\right)=1$$

and

(24) $$p\left(x\right)=\frac{1-\frac{1}{n}}{2\cdot (n-1)}=\frac{1}{2\cdot n}$$

For the next control qubit, we would obtain

$$p\left(x_{2}\right)=\frac{1-\frac{1}{n}}{4\cdot (n-1)}=\frac{1}{4\cdot n}$$

with

(25) $$p\left(x_{b}\right)=\frac{1-\frac{1}{n}}{2^{b}\cdot \left(n-1\right)}=\frac{1}{2^{b}\cdot n}$$

resulting in the sequence

$$p\left(x_{1}\right)=\frac{1}{2\cdot n},p\left(x_{2}\right)=\frac{1}{4\cdot n},p\left(x_{3}\right)=\frac{1}{8\cdot n},p\left(x_{4}\right)=\frac{1}{16\cdot n},\cdots ,p\left(x_{b}\right)=\frac{1}{2^{b}\cdot n}$$

After measuring the control qubit at step *b*, the probability of the marked state is (see Figure 23)

(26) $$p\left(marked_{b}\right)=\frac{\frac{1}{n}}{\frac{1}{n}+\left(n-1\right)\cdot p\left(x_{b}\right)}=\frac{\frac{1}{n}}{\frac{1}{n}+\left(n-1\right)\cdot \frac{1}{2^{b}\cdot n}}=\frac{2^{b}}{2^{b}-1+n}$$

and with the probability of measuring the control qubit at step *b*

(27) $$p\left(control_{1}\right)=\frac{1+n}{2\cdot n},\;\;\;p\left(control_{b}\right)=\frac{2^{b}+1+n}{2^{b}+2\cdot n}\;for\;b>1.$$

With the assumption of independence, measuring the control qubits in the sequence $b=1,b=2,b=3,\cdots ,b_{B}$

(28) $$p\left(control_{1},control_{2},\cdots ,control_{B}\right)=\prod_{j=1}^{B}p\left(control_{j}\right)$$

results in a low probability (see Figure 23). The assumption that “if *t* is lower (higher *b*;) than the determination of the desired states is better” is not correct. As a consequence, we can measure the sequential control qubits two times (b=2) before the task becomes not tractable.

## 8. Tree-like Structures

We want to increase the probability of measuring the correct units representing the ones in the answer vector and decrease the probability of measuring the zeros. For example, in a sparse code with *k* ones, *k* measurements of different ones reconstruct the binary answer vector and we cannot use the idea of applying Trugenberger amplification several times as indicated before. Instead, we can increase the probability of measuring a one by the introduced tree-like structure [44]. The tree-like hierarchical associative memory approach is based on aggregation of the neighboring units [44]. The aggregation is a Boolean OR-based transform for two or three neighboring weights of unit results resulting in a more dense memory, see Figure 24.

It was shown by computer experiments that the aggregation value between two and three is an optimal one [45]. The more dense memory is copied on top or the original memory. Depending on the number of units, we can repeat the process in which we aggregate groups of two to three neighboring groups of equal units. We can continue the process till we arrive in two different groups of different units, the number of possible different aggregated memories is logarithmic, with log(n−1). Since in our example only four units are present, we aggregate two units resulting in a memory of four units described by 2 identical units each.

**Figure 11:**
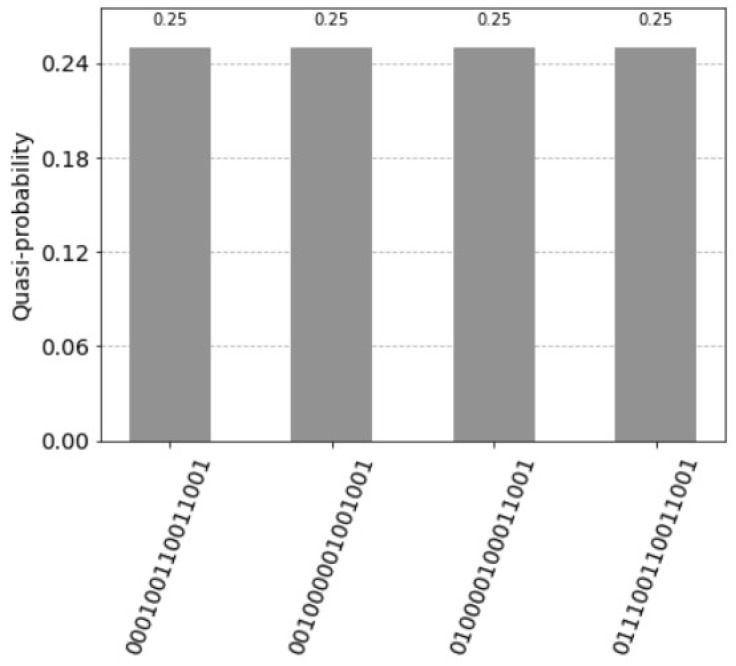
Four superposition states corresponding to the four units of the associative memory. The qubits 0 to 3 represent the query vector $x_{q}=\left(1,0,0,1\right)$, the qubits 4 to 7 the associative memory, the qubits 8 to 11 represent the count, the qubits 12 and 13 are the index qubits, and the control qubit 14 is zero. Note that the units are counted in the reverse order by the index qubits: 11 for the first unit, 10 for the third unit, 01 for second unit and 00 for the fourth unit.

**Figure 12:**
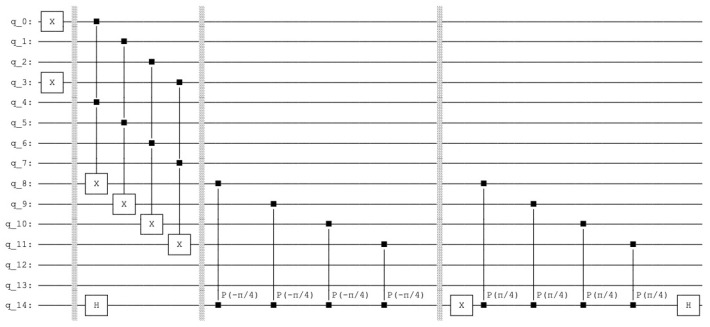
The quantum circuit that produces the *active phase*. The query and the amplification operations on the count qubits, the qubits 8 to 11. The control qubit 14.

**Figure 13:**
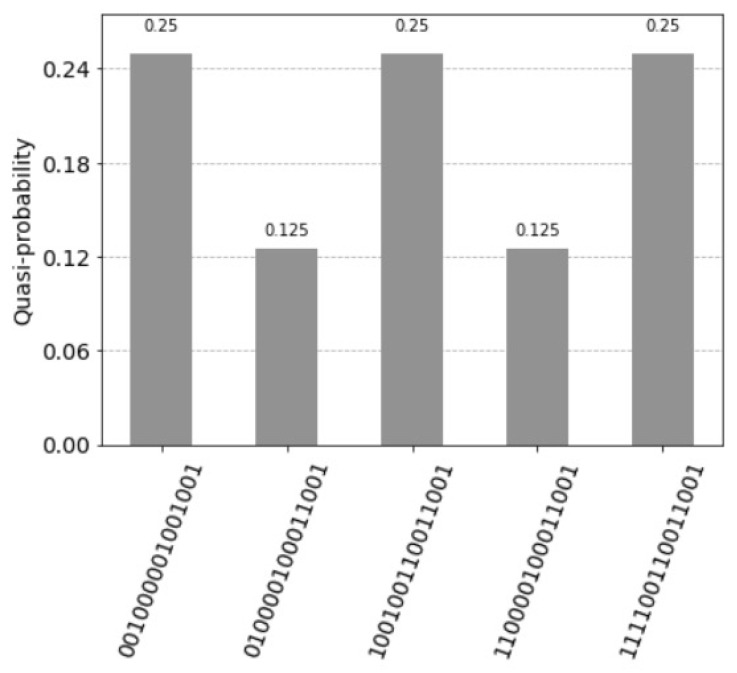
Five superposition states not equal to zero. The control qubit 14 equal to one indicates the firing of the units. The measured value is 0.625. The two probabilities 0.25 express the perfect match and the solution (1,0,0,1), indicated by the index qubits 12 and 13, with the values (11) for the first unit and (00) for the fourth unit. Note that the units are counted in the reverse order by the index qubits: (11) first unit, (10) for the second unit, (01) for third unit and (00) for the fourth unit. The control qubit 14 equal to zero indicates the units that do not fire. The measured value is 0.375. The probability 0.25 with the index qubits 12 and 13, with the value (01) for the third unit indicates the most dissimilar pattern (0,0,1,0).

**Figure 14:**
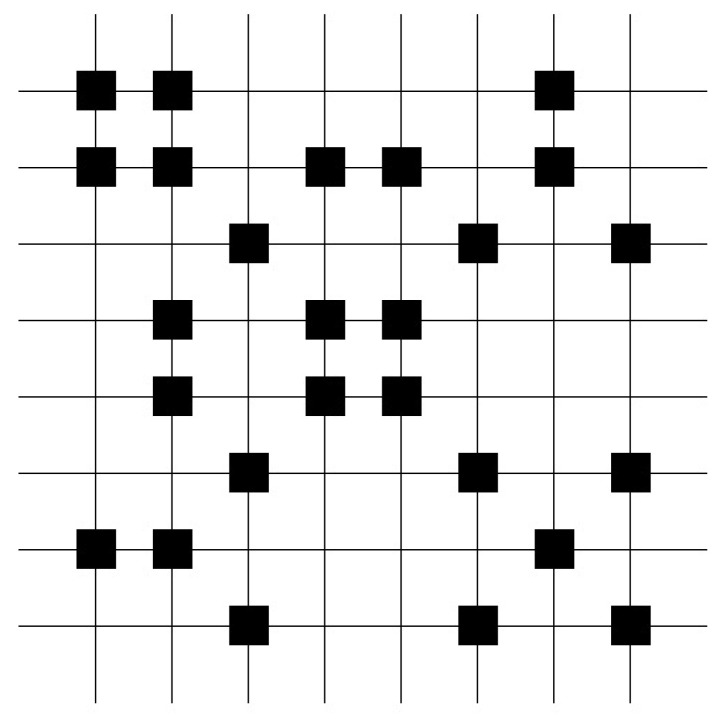
Weight matrix represented by eight units after learning the correlation of the three patterns $x_{1}=\left(1,1,0,0,0,0,1,0\right)$; $y_{1}=\left(1,1,0,0,0,0,1,0\right)$, $x_{2}=\left(0,1,0,1,1,0,0,0\right)$; $y_{2}=\left(0,1,0,1,1,0,0,0\right)$ and $x_{3}=\left(0,0,1,0,0,1,0,1\right)$; $y_{3}=\left(0,0,1,0,0,1,0,1\right)$. The learning is identical with the learning phase of the Lernmatrix.

**Figure 15:**
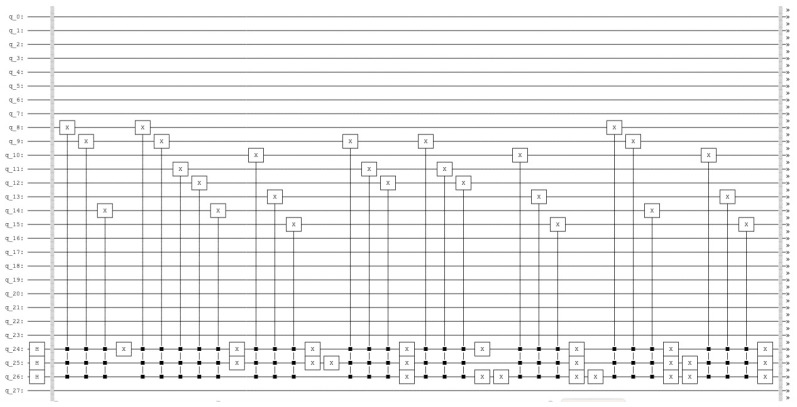
The quantum circuit that produces the *sleep phase*. The qubits 0 to 7 represent the query vector, the qubits 8 to 15 the associative memory, the qubits 16 to 23 represent the count and the qubits 24, 25 and 26 are the index qubits (8 states), and the qubit 27 is the control qubit.

**Figure 16:**
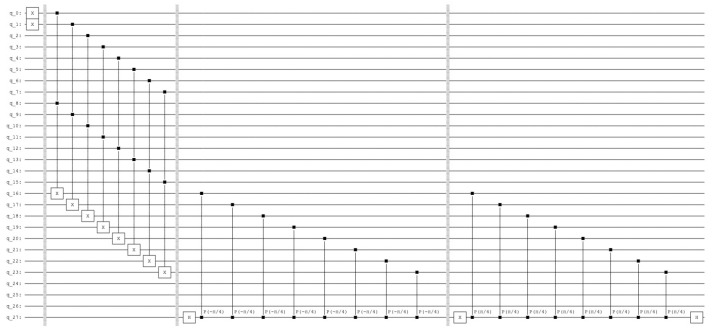
The quantum circuit that produces the *active phase*. The query and the amplification operations on the count qubits, the qubits 16 to 23 and the control qubit 27.

**Figure 17:**
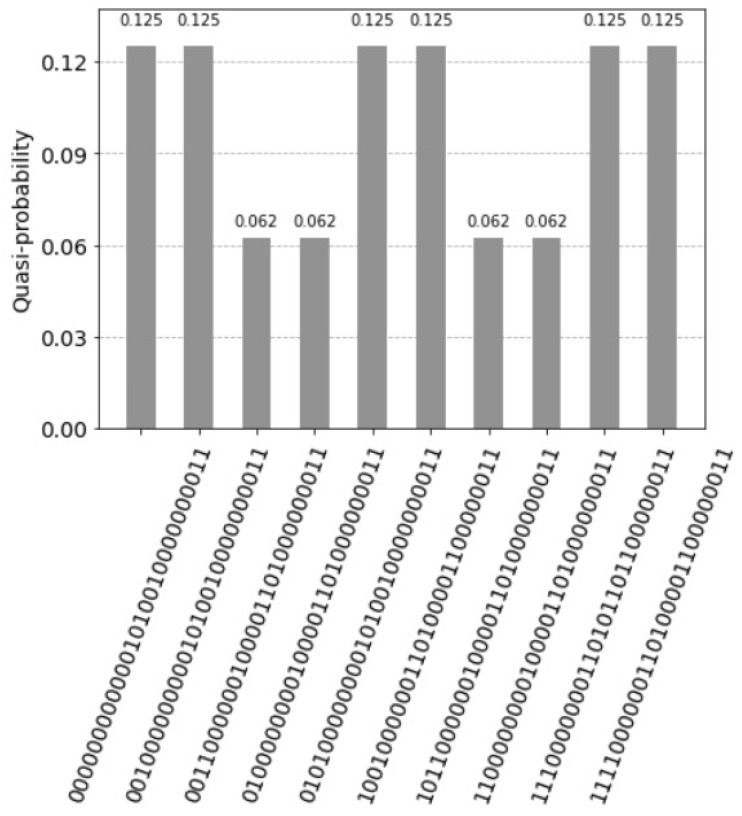
Teen superposition states not equal to zero. The qubits 24, 25 and 26 are the index qubits. Note that the units are counted in the reverse order by the index qubits: 111 first unit, 110 for the second unit, till 000 being the eight unit. The measured value for the control qubit 27 equal to one indicates the firing of the units. The measured value is just 0.5. This happens since the weight matrix is relatively small and not homogeneously filled. For the query vector $x_{q}=\left(1,1,0,0,0,0,0,0\right)$, the three values 0.125 indicate the answer vector (1,1,0,0,0,0,1,0) by the index qubits 24, 25 and 26; for the first unit with the value (111), the second unit (110) and seventh unit (001). The control qubit 27 equal to zero indicates the units that do not fire.

**Figure 18:**
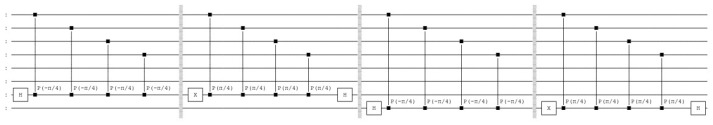
Circuit representing the application of the control qubit two times for the quantum circuit of Figure 10.

**Figure 19:**
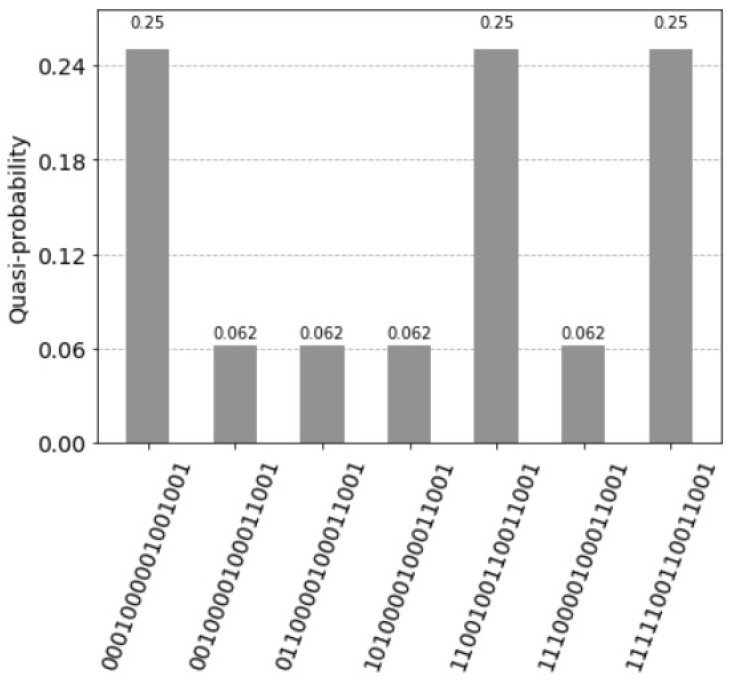
Seven superposition states not equal to zero. This is because the states with the former values 0.125 were divided into two values 0.125/2=0.0625 by the two control qubits. The first control qubit 15 equal to one indicates the firing of the units. The measured value is 0.625. After measuring the first control qubit equal to one, the measured value of the second control qubit 14 equal to one is 0.9. Assuming independence, the value of measuring the two control qubits with the value one is 0.5625=0.625·0.9. As before, the two values 0.25 indicate the perfect match and the solution (1,0,0,1) with the values of the index qubits 12 and 13: (11) for the first unit and (00) for the fourth unit.

**Figure 20:**
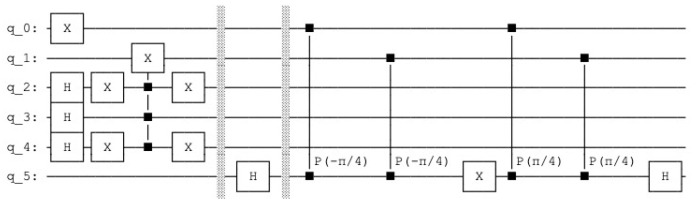
Assuming we have eight states indicated by the index qubit 2, 3 and 4, one marked state 010 has the count two, and the other seven state the count of one.

**Figure 21:**
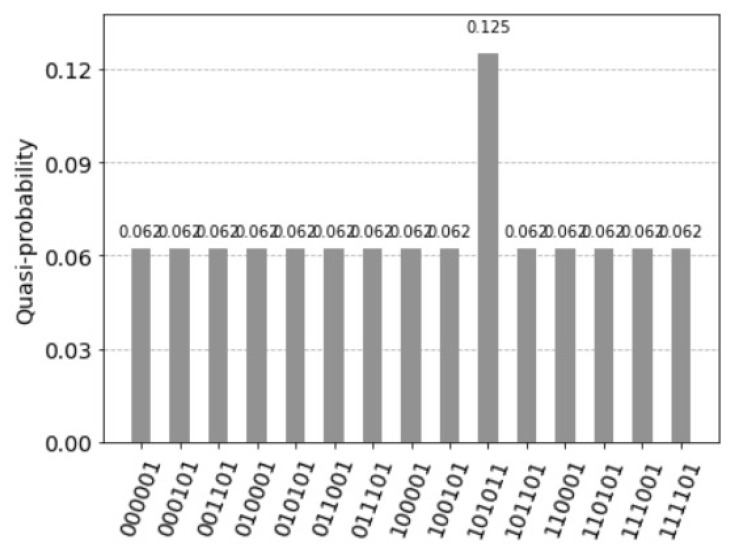
The resulting histogram of the measured qubits of one marked state with the count two, and the other seven state the count of one with applying the control qubit.

**Figure 22:**
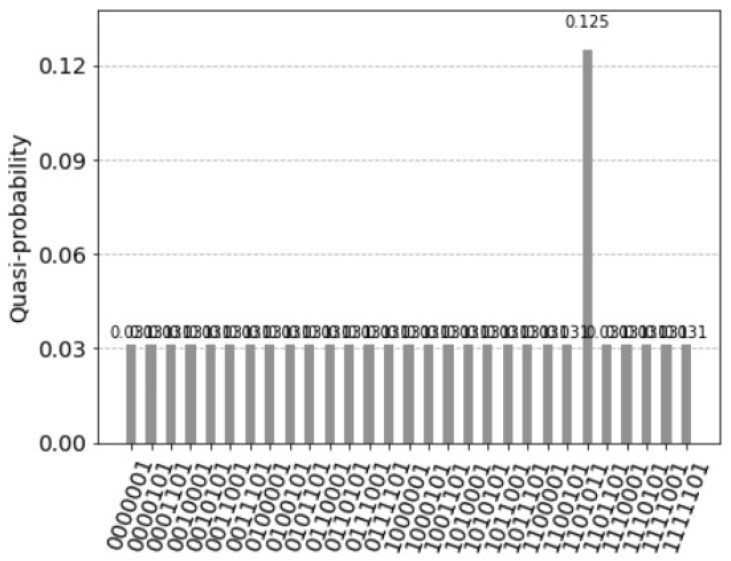
The resulting histogram of the measured qubits of one marked state with the count two, and the other seven state the count of one with applying the control qubit two times.

**Figure 23:**
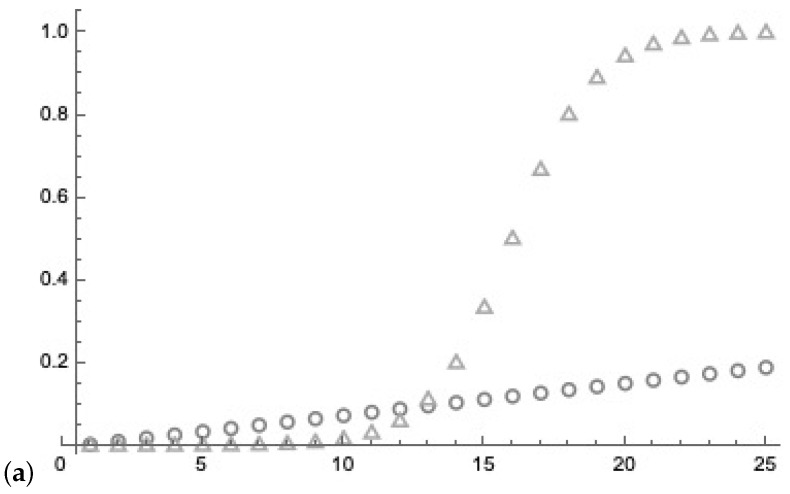

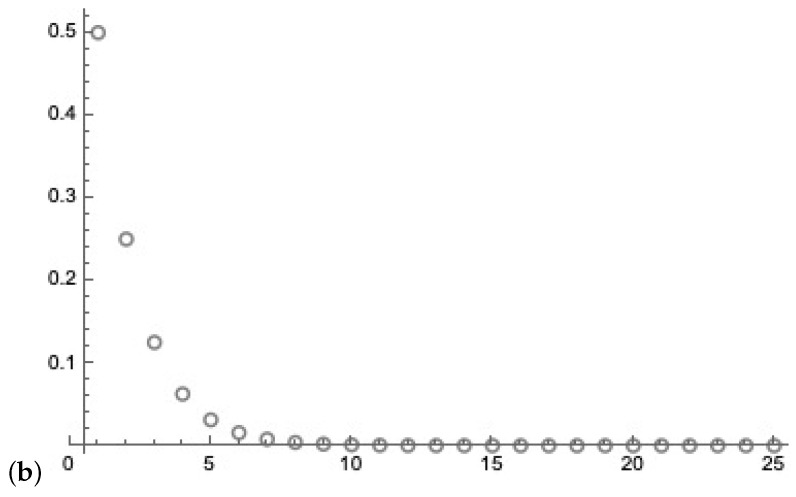
For $n=2^{16}$, the y-axis indicates the resulting probability. (**a**) Circles indicate the growth of the probability of the marked state related to the the number of steps of Grover’s amplification indicated by the x-axis. The triangles indicate the growth of the probability of the marked state using Trugenberger amplification with the x-axis indicating the number *b* of measurements assuming the control qubits are 1. (**b**) With the assumption of independence, measuring the control qubits in the sequence $b=1,b=2,b=3,\cdots ,b_{B}$ results in a low probability indicated by the circles. The x-axis indicates the number measurements *b* of the control qubits. As a consequence, we can measure the sequential control qubits two times before the task becomes not tractable.

**Figure 24:**
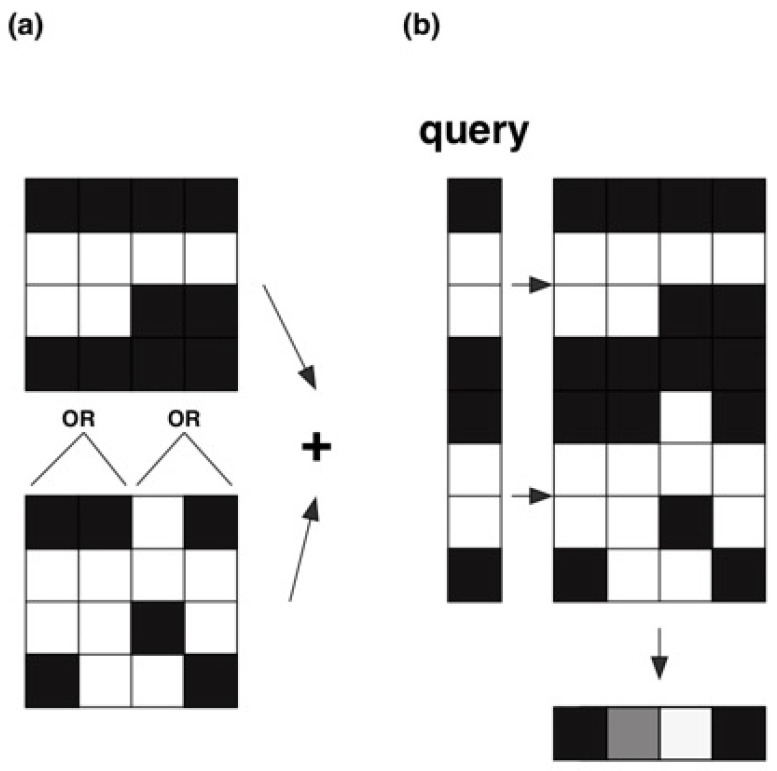
(**a**) In our example, we store three patterns, $x_{1}=\left(1,0,0,1\right)$, $y_{1}=\left(1,0,0,1\right)$; $x_{2}=\left(1,0,0,0\right)$, $y_{2}=\left(0,1,0,0\right)$ and $x_{3}=\left(0,0,1,0\right)$, $y_{3}=0,0,1,0)$, and the query vector is $x_{q}=\left(1,0,0,1\right)$. (**b**) The aggregation is a Boolean OR-based transform for two neighboring weights of units results resulting in a more dense memory with $x_{q}=\left(1,0,0,1,1,0,0,1\right)$.

**Figure 25:**
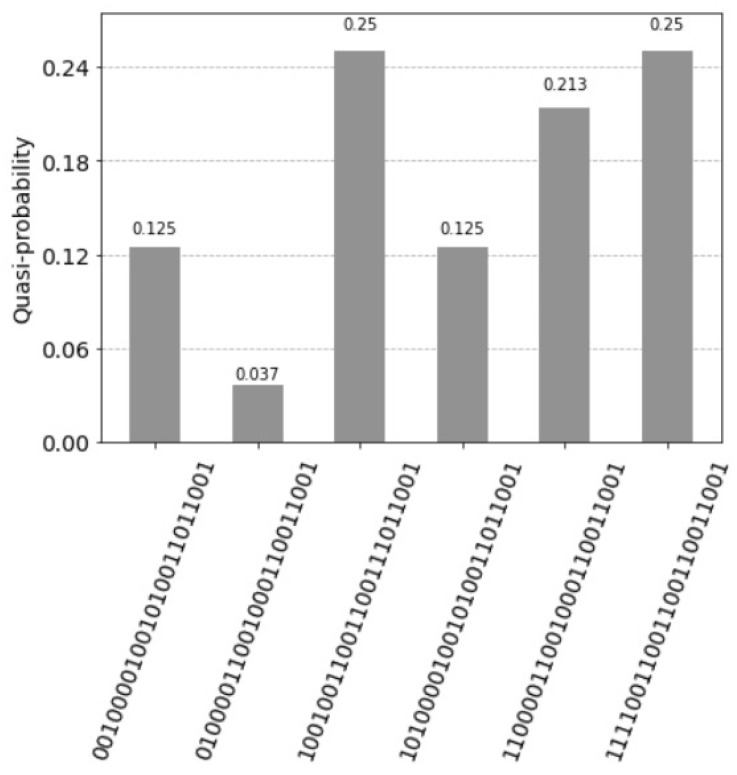
Five superposition states not equal to zero. The measured probability (control qubit equal to one) indicates the firing of the units is 0.838, the measured probability values are 0.213, 0.125 and 0.25.

The query vector is composed of log(n−1) concatenated copies of the original query vector, in our example $x_{q}=\left(1,0,0,1,1,0,0,1\right)$. We apply controlled phase operation with N=4 with $count_{j}\leq 4$, see Figure 24 and Appendix B. The measured probability (control qubit=1) indicating a firing of the units is 0.838 and there are six states not equal to zero, see Figure 25 and compare with Figure 11.

## 9. Costs

We cannot clone an arbitrary quantum state; however, it was proposed that a quantum state can be probabilistic cloned up to a mirror modular transformation [14]. In an alternative approach, we prepare a set of quantum Lernmatrices in superposition. This preparation requires a great deal of time and we name it the *sleep phase*. The cost of storing $L=\left(\ln2\right)\left(n^{2}/k^{2}\right)$ patterns in *n* units with $k=\log_{2}\left(n/4\right)$ in a Lernmatrix and consequently the quantum Lernmatrix is $O(n^{2})$  [18,19]. On the other hand, in the *active phase*, the query operation is extremely fast.

### Query Cost of Quantum Lernmatrix

**Figure 26:**
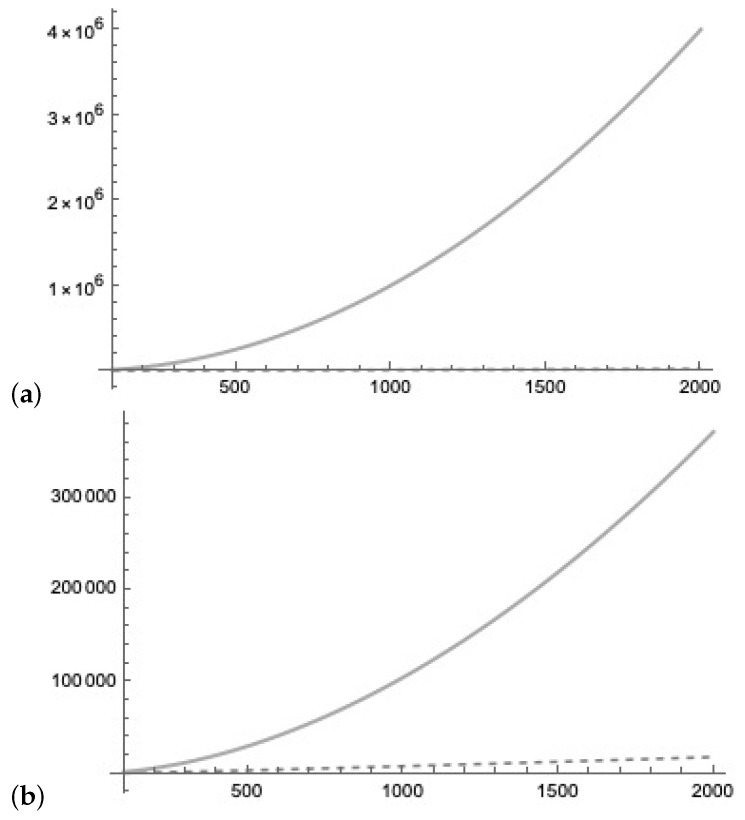
(**a**) We compare the cost of $k=log_{2}\left(n/4\right)$ queries to the quantum Lernmatrix (representing the weight matrix of the size n×n), O(k·n) (dashed line) to the cost of a classical Lernmatrix of the size n×n, $O(n^{2})$. (**b**) We compare the cost of *k* queries to the quantum Lernmatrix (representing the weight matrix of the size n×n), O(k·n) with cost O(k·n) (dashed line) to Grover’s amplification algorithm on a list of *L* vectors of dimension *n* with cost $O(n\cdot \sqrt{L})$.

During the *active phase*, the quantum Lernmatrices are sampled with minimal costs in time. In Figure 26a, we compare the query cost of *k* queries of the quantum Lernmatrix representing the weight matrix of the size n×n to the cost of a classical Lernmatrix of the size n×n, which are

$$O\left(\log\left(n\right)\cdot n\right)<O\left(n^{2}\right).$$

In Figure 26b, we compare the query cost of *k* queries of the quantum Lernmatrix representing the weight matrix of the size n×n to Grover’s amplification algorithm on a list of *L* vectors of dimension *n*

$$O\left(n\cdot \sqrt{L}\right)=O\left(\frac{n^{2}}{\log(n)}\right).$$

## 10. Conclusions

We introduced a quantum Lernmatrix based on the Monte Carlo Lernmatrix and preformed experiments using *qiskit* as a proof of concept for future quantum associative memories. We proposed a tree-like structure that increases the measured value for the control qubit indicating a firing of the units. Our approach does not solve the input destruction problem but gives a hint how to deal with it. We represent the preparation costs and the query time by two phases.

The cost of the *sleep phase* and the *active phase* are the same as one of a conventional associative memory $O(n^{2})$. We assume that in the *sleep phase* we have enough time to prepare several quantum Lernmatrices in superposition. The quantum Lernmatrices are kept in superposition until they are queried in the *active phase*. Each of the copies of the quantum Lernmatrix can be queried only once. We argue that the advantage to conventional associative memories is present in the *active phase* were the fast determination of information O(log(n)·n) is essential by the use of quantum Lernmatrices in superposition compared to the cost of the classical Lernmatrix $O(n^{2})$.

## Data Availability

Not appliable.

## Appendix A. Quantum Lernmatrix

In our architecture the qubits 0 to 3 represent the query vector, the qubits 4 to 7 the associative memory, the qubits 8 to 11 represent the count and the qubits 12 and 13 are the index qubits, the qubit 14 is the control qubit. The count operation is done by the ccX gate (see Figure 10 and Figure 12)

qc = QuantumCircuit(15)
#0-3 query
#4-7 data
#8-11 count
#Index Pointer
#12-13
#Aux
#14

#Sleep Phase
#Index Pointer
qc.h(12)
qc.h(13)
qc.barrier()
#1st weights
qc.ccx(12,13,4)
qc.ccx(12,13,7)
qc.barrier()
#2th weights
qc.x(12)
qc.ccx(12,13,4)
qc.x(12)
qc.barrier()
#3th weights
qc.x(13)
qc.ccx(12,13,6)
qc.x(13)
qc.barrier()
#4th weights
qc.x(12)
qc.x(13)
qc.ccx(12,13,4)
qc.ccx(12,13,7)
qc.x(13)
qc.x(12)
qc.barrier()

#Active Phase
#query
qc.x(0)
qc.x(3)
qc.barrier()
qc.ccx(0,4,8)
qc.ccx(1,5,9)
qc.ccx(2,6,10)
qc.ccx(3,7,11)
#Dividing
qc.h(14)
qc.barrier()
#Marking
qc.cp(-pi/4,8,14)
qc.cp(-pi/4,9,14)
qc.cp(-pi/4,10,14)
qc.cp(-pi/4,11,14)
qc.barrier()
qc.x(14)
qc.cp(pi/4,8,14)
qc.cp(pi/4,9,14)
qc.cp(pi/4,10,14)
qc.cp(pi/4,11,14)
qc.h(14)
qc.draw(fold=110)

## Appendix B. Quantum Tree-like Lernmatrix

qc = QuantumCircuit(23)
#0-3 query
#4-7 data aggregated
#8-11 data
#12-19 count
#Index Pointer
#20-21
#Aux
#22

#Sleep Phase
#Index Pointer
qc.h(20)
qc.h(21)
#1st weights
#OR Aggregated
qc.barrier()
qc.ccx(20,21,4)
qc.ccx(20,21,7)
#Original
qc.barrier()
qc.ccx(20,21,8)
qc.ccx(20,21,11)
#2th weights
qc.x(20)
#OR Aggregated
qc.barrier()
qc.ccx(20,21,4)
qc.ccx(20,21,7)
#Original
qc.barrier()
qc.ccx(20,21,8)
qc.x(20)
#3th weights
qc.x(21)
#OR Aggregated
qc.barrier()
qc.ccx(20,21,4)
qc.ccx(20,21,6)
qc.ccx(20,21,7)
#Original
qc.barrier()
qc.ccx(20,21,10)
qc.x(21)
#4th weights
qc.x(20)
qc.x(21)
#OR Aggregated
qc.barrier()
qc.ccx(20,21,4)
qc.ccx(20,21,6)
qc.ccx(20,21,7)
#Original
qc.barrier()
qc.ccx(20,21,8)
qc.ccx(20,21,11)
qc.x(21)
qc.x(20)

#Active Phase
#query
qc.barrier()
qc.x(0)
qc.x(3)
qc.barrier()
#query, counting
#OR Aggregated
qc.ccx(0,4,12)
qc.ccx(1,5,13)
qc.ccx(2,6,14)
qc.ccx(3,7,15)
#Original
qc.ccx(0,8,16)
qc.ccx(1,9,17)
qc.ccx(2,10,18)
qc.ccx(3,11,19)
#Dividing
qc.barrier()
qc.h(22)
#Marking
qc.barrier()
qc.cp(-pi/8,12,22)
qc.cp(-pi/8,13,22)
qc.cp(-pi/8,14,22)
qc.cp(-pi/8,15,22)
qc.cp(-pi/8,16,22)
qc.cp(-pi/8,17,22)
qc.cp(-pi/8,18,22)
qc.cp(-pi/8,19,22)
qc.barrier()
qc.x(22)
qc.cp(pi/8,12,22)
qc.cp(pi/8,13,22)
qc.cp(pi/8,14,22)
qc.cp(pi/8,15,22)
qc.cp(pi/8,16,22)
qc.cp(pi/8,17,22)
qc.cp(pi/8,18,22)
qc.cp(pi/8,19,22)
qc.barrier()
qc.h(22)
qc.draw()
